# β-Naphthoflavone and Ethanol Reverse Mitochondrial Dysfunction in A Parkinsonian Model of Neurodegeneration

**DOI:** 10.3390/ijms21113955

**Published:** 2020-05-31

**Authors:** Jesus Fernandez-Abascal, Elda Chiaino, Maria Frosini, Gavin P. Davey, Massimo Valoti

**Affiliations:** 1 Dipartimento di Scienze della Vita, Università di Siena, 53100 Siena, Italy; maria.frosini@unisi.it (M.F.); massimo.valoti@unisi.it (M.V.); chiaino@student.unisi.it (E.C.); 2 Department of Neurodegeneration and Systems Biology, School of Biochemistry and Immunology, Trinity College Dublin, D02 PN40 Dublin, Ireland; gdavey@tcd.ie

**Keywords:** Cytochrome P-450 system, CYP induction, CYP 2D6, CYP 2E1, mitochondrial kinetics, MPP^+^ toxicity, neurodegeneration, neuroprotection

## Abstract

The 1-methyl-4-phenylpyridinium (MPP^+^) is a parkinsonian-inducing toxin that promotes neurodegeneration of dopaminergic cells by directly targeting complex I of mitochondria. Recently, it was reported that some Cytochrome P450 (CYP) isoforms, such as CYP 2D6 or 2E1, may be involved in the development of this neurodegenerative disease. In order to study a possible role for CYP induction in neurorepair, we designed an in vitro model where undifferentiated neuroblastoma SH-SY5Y cells were treated with the CYP inducers β-naphthoflavone (βNF) and ethanol (EtOH) before and during exposure to the parkinsonian neurotoxin, MPP^+^. The toxic effect of MPP^+^ in cell viability was rescued with both βNF and EtOH treatments. We also report that this was due to a decrease in reactive oxygen species (ROS) production, restoration of mitochondrial fusion kinetics, and mitochondrial membrane potential. These treatments also protected complex I activity against the inhibitory effects caused by MPP^+^, suggesting a possible neuroprotective role for CYP inducers. These results bring new insights into the possible role of CYP isoenzymes in xenobiotic clearance and central nervous system homeostasis.

## 1. Introduction

Degeneration of dopaminergic neurons in the substantia nigra is a main feature of Parkinson’s disease (PD). Genetic and environmental factors are known to give rise to differential proteostatic states in the brain. Additionally, cell specific energy metabolism defects and reactive oxygen species (ROS) production can enhance neurodegeneration rates [1]. ROS can also be generated by exposure to environmental xenobiotics or drug metabolism. In addition, genetic predisposition contributes to selective neurodegeneration of these cells [2,3,4,5]. High oxidative stress and disruptors of mitochondrial membrane potential (ψ_m_) lead to a change in morphology and alteration in mitochondrial fusion-fission dynamics [6]. These events disrupt the overall mitochondrial homeostasis, promoting apoptosis, the release of pro-apoptotic factors, and contributing to neurodegeneration in PD and other diseases [7]. In this context, xenobiotics have emerged as one important source of oxidative stress that may lead to mitochondrial dysfunction in the brain [8,9].

A mechanism by which the cells clear xenobiotics is by the Cytochrome P-450 system (CYP), which metabolize a wide variety of molecules [10]. In the brain, CYP represents a 0.5–2% of the total amount of CYP found in the liver, but it still plays an important role in the metabolism of drugs and some endogenous compounds in the central nervous system (CNS) [11]. This superfamily has several isoforms with specific expression patterns depending on the brain area and the cell type [12,13]. In particular, the isoform CYP 2D6 has been related with the development of PD due to its ability to metabolize several xenobiotics in dopaminergic cells and other areas [14]. Moreover, in dopaminergic cells, the induction of CYP 2E1 by nicotine or coffee has been related with less susceptibility to PD [15,16]. However, the contributions of CYPs isoforms to neurodegeneration and neuroprotection toward xenobiotic insult are still poorly understood. 

Induction of CYP isoforms have been generally used for the study of drug metabolism and neuroprotection in vivo and in vitro [11]. Ethanol (EtOH) and β-naphthoflavone (βNF) are two well-known inducers of CYP isoforms [17,18,19]. We previously reported that both compounds promote the induction of CYP 2D6 and 2E1, and that CYP 2D6 can be localized in mitochondria in SH-SY5Y cells [20]. The objective of the present study is to elucidate whether treatments with both inducers protect mitochondria towards the neurotoxic effect of MPP^+^. Our results suggest that, in parallel with induction of CYP isoforms 2D6 and 2E1, the two compounds reverse the mitochondrial impairment promoted by MPP^+^. 

## 2. Results

### 2.1. βNF and EtOH Treatment Reduce the Loss of Cell Viability by MPP^+^

**Figure 1:**
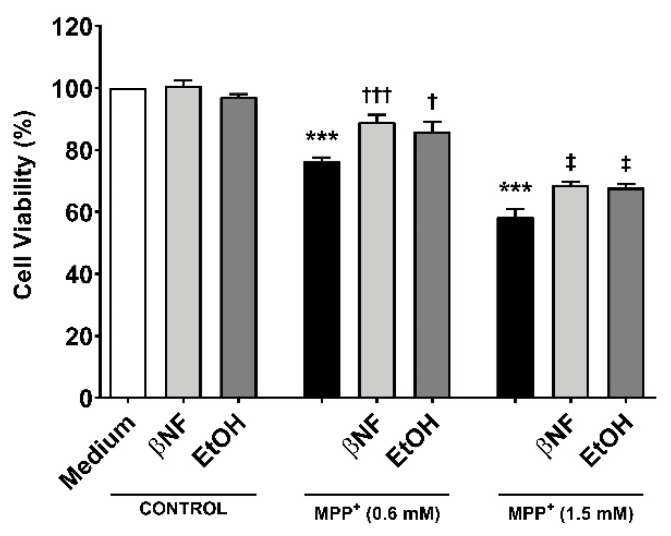
MPP^+^ toxicity is reduced by β-naphthoflavone (βNF) and ethanol (EtOH) treatment. SH-SY5Y cells treated with MPP^+^ (0.6 mM and 1.5 mM) caused a decrease in cell viability. This was calculated measuring the absorbance of formazan generated by metabolism of MTT (see methods). However, the treatment with CYPs inducers partially reversed it for both MPP^+^ concentrations (see Table 1 for treatment details). Columns represent mean ± SEM of independent cell culture preparations (*n* ≥ 3; usually ~8), each one containing at least three biological replicates. Data are represented as percentage of control samples, whose absorbance was taken as 100% viability. *** *p* < 0.001 vs. βNF or EtOH; ^†††^ *p* < 0.001, ^†^ *p* < 0.05 vs. MPP^+^ 0.6 mM; ^‡^ *p* < 0.05 vs. MPP^+^ 1.5 mM, two-way ANOVA followed by Tukey post-test.

To study whether the induction of CYPs by βNF and EtOH can protect towards MPP^+^-induced toxicity, two different concentrations of the toxin were used in presence or absence of the two inducers (Table 1). Under this treatment protocol, we assessed cell viability based on the MTT-to-formazan metabolic activity [21]. Low (0.6 mM) and high (1.5 mM) concentrations of MPP^+^ promoted a decrease in cell viability of 24% and 42%, respectively (Figure 1). When cells were treated with βNF prior and during exposure to MPP^+^ itself (both concentrations), a significant recovery of ~17% in cell viability was observed. EtOH showed similar results, as ~15% recovery was in fact found at both MPP^+^ concentrations. These results suggest that βNF and EtOH may contribute to a better cellular protection against MPP^+^, possibly via CYP induction.

**Figure 2:**
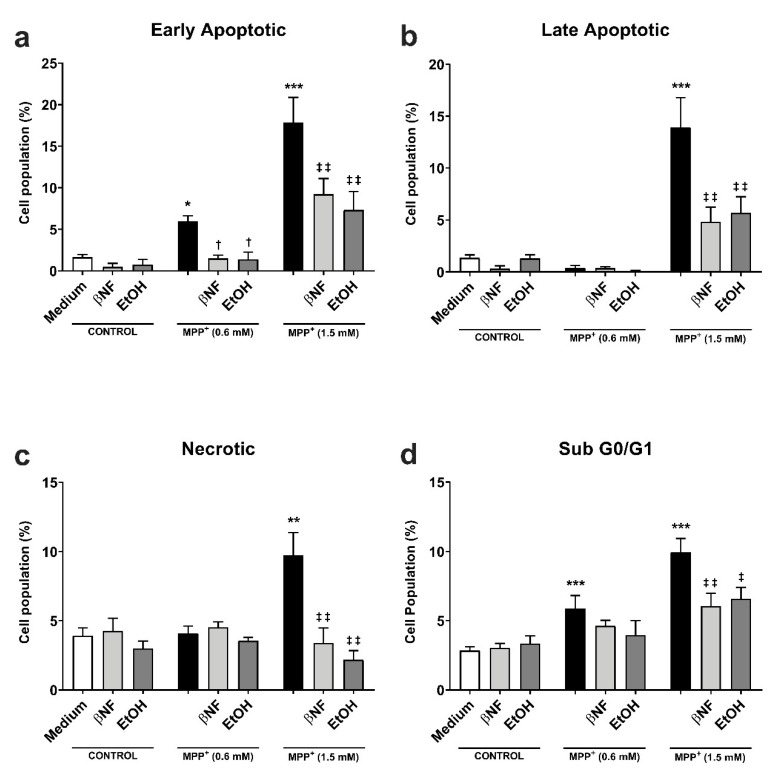
βNF and EtOH treatments reduced MPP^+^-mediated apoptosis. (**a**–**c**) Cell viability assay with Annexin V/PI double staining. Both MPP^+^ treatments (0.6 mM and 1.5 mM) were able to increase the early apoptotic population (**a**), while only MPP^+^ (1.5 mM) increased the late apoptotic (**b**) and necrotic populations (**c**). The treatments with inducers (see Table 1) were able to rescue the apoptosis promoted by MPP^+^. **d:** MPP^+^ promotes an increase in the percentage of hypodiploid (sub G0/G1) cells, which was reported by the increase of PI-stained DNA content. The treatment with inducers (see Table 1) partially rescued the toxic effect of MPP^+^. Data are reported as mean ± SEM of at independent cell culture preparations (*n* ≥ 3; usually ~10). *** *p* < 0.001, ** *p* < 0.01, * *p* < 0.05 vs. Medium; ^†^ *p* < 0.05 vs. MPP^+^ 0.6 mM; ^‡‡^ *p* < 0.01, ^‡^ *p* < 0.05 vs. MPP^+^ 1.5 mM, two-way ANOVA followed by Tukey post-test.

The protective effect of CYP induction obtained in the MTT experiments was further investigated by analyzing the formation of apoptotic/necrotic cells with Annexin V/ propidium iodide (AV/PI) staining. The early apoptotic cell population increased in an MPP^+^-related concentration manner (+6% MPP^+^ 0.6 mM; +18% MPP^+^ 1.5 mM) (Figure 2a). The treatments with βNF and EtOH significantly reverted the toxic effect of both concentrations of MPP^+^. βNF restored this population to values similar to control samples (1.5%) in βNF + MPP^+^ (0.6 mM) treatments, while it reduced the early apoptotic population to 9% in βNF + MPP^+^ (1.5 mM) treatments. On the other hand, EtOH showed similar effectiveness, as it reduced AV+/PI- cells to control values (1.4%) in EtOH + MPP^+^ (0.6 mM), while in treatments with EtOH + MPP^+^ (1.5 mM), it significantly decreased it to 7%. The same protective effect was observed in late apoptotic cells with MPP^+^ (1.5 mM) treatments, as only this concentration increased the AV+/PI+ cells by 14% (Figure 2b). In this case, the treatment with βNF and EtOH significantly reduced it to 4.8% and 5.7%, respectively. Finally, the necrotic cells reached the value of 7% upon the highest MPP^+^ concentration used, while both CYP inducers significantly reduced them to values comparable to controls (Figure 2c). 

The effects of MPP^+^ on the cell cycle were also investigated. The neurotoxin induced a concentration-dependent rise in sub-G0/G1 hypodiploid cells, typical of apoptotic-mediated cell death. In particular, the subG0/G1 group was significantly increased at both low and high MPP^+^ concentrations (6% and 10%, respectively) when compared to either βNF or EtOH groups (Figure 2d). The treatment with the two inducers reverted this effect only in high concentration of MPP^+^, although a similar trend could be observed for low concentrations: βNF + MPP^+^ (0.6 mM), 4.6%; βNF + MPP^+^ (1.5 mM), 6%; EtOH + MPP^+^ (0.6 mM), 4%; EtOH + MPP^+^ (1.5 mM), 6.6%. These data suggest that βNF and EtOH decrease the apoptotic-mediated cell death caused by MPP^+^.

### 2.2. MPP^+^-Related ROS Production is Decreased with CYP Inducers

**Figure 3:**
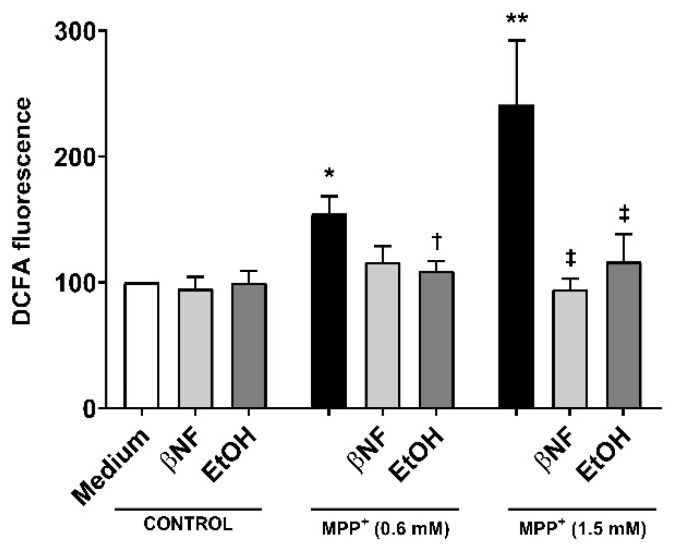
Reactive oxygen species (ROS) production by MPP^+^ was decreased in cells treated with CYP inducers. MPP^+^ treatments increased the production of ROS in SH-SY5Y cells at both concentrations. When cells were treated with βNF or EtOH prior and during exposure to MPP^+^ (see Table 1 for treatment details), ROS production was decreased. Columns represent the percentage related to control (medium) samples of DCFA fluorescence, and data are presented as mean ± SEM of independent cell culture preparations (*n* ≥ 5). ** *p* < 0.01, * *p* < 0.05 vs. βNF or EtOH; ^†^ *p* < 0.05 vs. MPP^+^ 0.6 mM; ^‡^ *p* < 0.05 vs. MPP^+^ 1.5 mM, two-way ANOVA followed by Tukey post-test.

The treatments with EtOH and βNF showed that, during the exposure time to MPP^+^, the cells no longer underwent late apoptosis or necrosis, and only toxic effects could be observed in early apoptotic populations. This suggested that the toxicity generated by MPP^+^ was reduced by EtOH and βNF, thus the production of the initial triggers of apoptosis might also have been decreased. To pursue this idea, we measured ROS formation and found that MPP^+^ (0.6 mM and 1.5 mM) increased DCFA fluorescence by 54% and 141%, respectively (Figure 3). Interestingly, when cells were treated with the inducers, the ROS production was significantly lower in EtOH + MPP^+^ (0.6 mM and 1.5 mM: 9% and 16% increase compared to control samples, respectively; or −83% and −87% compared to MPP^+^ samples, respectively). In βNF + MPP^+^ (0.6 mM), a decrease in ROS production was also observed, although not significant (15% increase compared to control samples; or −72% compared to MPP^+^ sample), while in βNF + MPP^+^ (1.5 mM), the ROS production was significantly lower (−6% compared to control samples; or −104% compared to MPP^+^ sample). These results suggest that treatment with both inducers prevents cells from undergoing apoptosis, probably by reducing ROS formation caused by the exposure to a mitochondrial-targeted toxin.

### 2.3. Mitochondrial Fusion Kinetics Is Restored Under βNF and EtOH Treatment

The mitochondria is the major target for MPP^+^, and its fusion kinetics represent important data to evaluate the integrity of the cells, since it can work as an indicator of the first stage of apoptosis. To further investigate the apoptotic-inducing effects of MPP^+^, the mitochondrial fusion dynamics were studied with MPP^+^ (0.6 mM) treatments only in order to discard false positive results from late apoptotic or necrotic cells that would be found with MPP^+^ (1.5 mM) treatments. 

**Figure 4:**
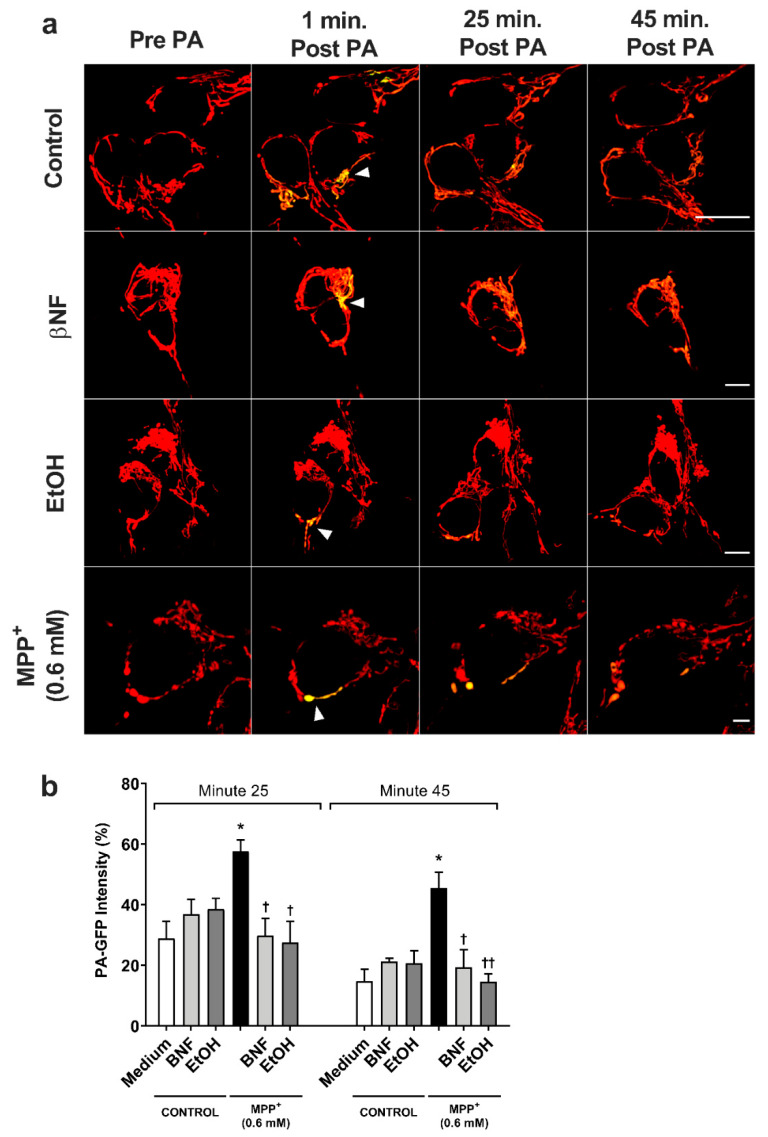
MPP^+^-induced damage to mitochondrial fusion dynamics in SH-SY5Y cells was reversed by βNF and EtOH. (**a**) Representative confocal images of SH-SY5Y showing the PA-GFP fluorescent spread over the time. Pictures are a combination of red channel for the mitochondrial reporter and the green channel for PA-GFP. Arrowheads point the photo-activated area. Scale bar: 10 µm. (**b**) PA-GFP percentage intensity in the photo-activated area at minutes 25 and 45 post-photoactivation. Mitochondrial fusion dynamics after treatment with βNF + MPP^+^ (0.6 mM) or EtOH + MPP^+^ (0.6 mM) returned to control values. Intensity in the green channel before photo-activation was taken as 0%, while intensity in one-minute post photo-activation was considered as 100% intensity. Columns represent mean ± SEM of independent cell culture preparations (*n* ≥ 3). * *p* < 0.05 vs. medium; ^††^ *p* < 0.01, ^†^ *p* < 0.05 vs. MPP^+^ 0.6 mM, two-way ANOVA followed by Tukey post-test.

For the MPP^+^ (0.6 mM) control, the fluorescent intensity of the photo-activated area decreased from 100% to 58% in 25 minutes (Figure 4 and Supplementary Figure S1), while control condition showed higher fusion dynamics (29% intensity at 25 min). In both βNF + MPP^+^ (0.6 mM) and EtOH + MPP^+^ (0.6 mM) treatments, the intensity of the photo-activated area showed a higher rate of decrease than in MPP^+^ (0.6 mM) control. Indeed, the results showed that 25 minutes post-activation, the plasmid containing the photo-activatable green fluorescent protein (PA-GFP) intensity decreased from 100% to 30% for βNF + MPP^+^ (0.6 mM) and to 28% for EtOH MPP^+^ (0.6 mM) treatment, values similar to control samples. Moreover, at minute 45 post-activation, this intensity decreased until 19% for βNF + MPP^+^ (0.6 mM) and to 15% for EtOH + MPP^+^ (0.6 mM) treatment, while MPP^+^ (0.6 mM) only reached a 46% intensity. Under our experimental conditions, both treatments were able to avoid the mitochondrial instability promoted by MPP^+^, resulting in normal fusion dynamics.

### 2.4. Disruption of ψ_m_ by MPP^+^ Is Avoided with CYP-Inducers Treatments

**Figure 5:**
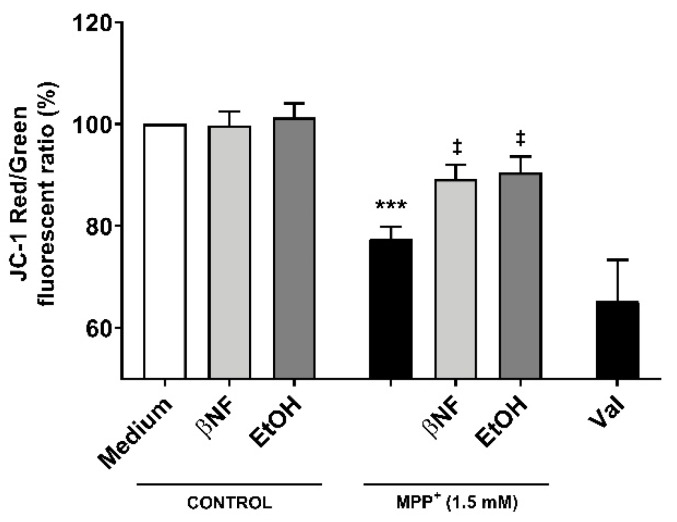
Changes in ψ_m_ by MPP^+^ were restored by βNF and EtOH treatments. SH-SY5Y cells were treated with MPP^+^ (1.5 mM) and JC-1 was used to study the ψ_m_. In control conditions, JC-1 accumulated inside the mitochondria, emitting red fluorescence. When the Ψ_m_ was disrupted, JC-1 accumulated in cytoplasm, emitting green fluorescence. Valinomycin (Val), which permeabilizes the mitochondrial membrane to potassium, dissipated the mitochondrial electrochemical potential and was used as a positive control to prevent JC-1 aggregation. Data represent the red/green ratio of treated cells and are depicted as a percentage of control samples of independent cell culture preparations (*n* ≥ 3). *** *p* < 0.001 vs. βNF or EtOH; ^‡^ *p* < 0.05 vs. MPP^+^ 1.5 mM, two-way ANOVA followed by Tukey post-test.

Because the restoration of mitochondrial kinetics is an indicator of proper mitochondrial functioning, we then assessed whether the treatments with inducers would also be able to restore the ψ_m_. The red/green fluorescent intensity ratio observed when cells were treated with MPP^+^ (1.5 mM) was 32.5% smaller than control samples, indicating a higher accumulation of 5,5′,6,6′-tetrachloro-1,1′,3,3′-tetraethyl-benzimidazolocarbocyanine iodide (JC-1) monomers (Figure 5). Both βNF + MPP^+^ (1.5 mM) and EtOH + MPP^+^ (1.5 mM) treatments were able to partially reverse this decrease and showed a significant recover (89% and 90%, respectively) in the red/green ratio. An important decrease in JC-1 aggregates in valynomicine control samples confirmed that the observed changes were due to dissipation of the electrochemical potential. These data suggest that the possible protection exerted by the treatments affects mechanisms that are upstream to any impairment of mitochondrial functioning promoted by MPP^+^ toxicity.

### 2.5. Treatments with βNF and EtOH Avoid Decrease of Mitochondrial Complex I Activity by MPP^+^

**Figure 6:**
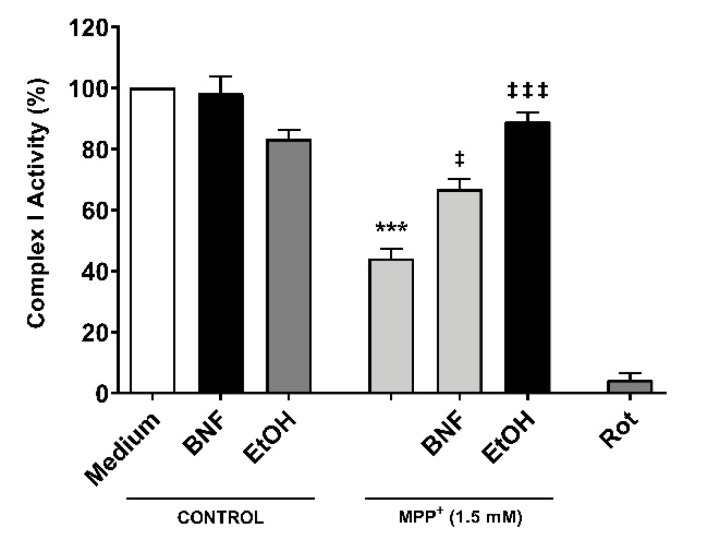
Reduction of mitochondrial complex I activity by MPP^+^ was partially avoided with βNF and EtOH pre-treatments. SH-SY5Y cells were pre-incubated with either inducer for 48 hours prior exposure to MPP^+^ in the moment of data acquisition. Rotenone (Rot) was used as control for NADH reduction by complex I. Columns represent the percentage of complex I activity of each group (see Methods) and represent the mean ± SEM of independent cell culture preparations (*n* = 3). *** *p* < 0.001 vs. βNF or EtOH; ^‡‡‡^ *p* < 0.001, ^‡^ *p* < 0.05, vs. MPP^+^ 1.5 mM, two-way ANOVA followed by Tukey post-test.

In order to assess whether CYP inducers protect the correct functioning of mitochondria from MPP^+^, a complex I activity assay was carried out. Cells were pre-incubated for 48 hours with βNF or EtOH at reported concentrations, then treated with MPP^+^ (1.5 mM) at the moment of data acquisition. We observed a decrease in the complex I activity of 56% compared to control (Figure 6). The inhibition of complex I activity obtained by rotenone confirmed that complex I was involved in the NADH reduction measured in this assay. Both pre-treatments with βNF and EtOH significantly reduced the toxic effect of MPP^+^, showing only 33% and 11% decreases of complex I activity, respectively. These data suggest that CYP induction predisposes the cells to be more efficient in avoiding the toxicity produced by MPP^+^, and that this protection occurs before the toxin disrupts the mitochondrial complex I activity. Overall, the presented data suggest that βNF and EtOH, possibly by inducing CYPs, protect the SH-SY5Y cells against toxic insult by MPP^+^.

## 3. Discussion

Exposure to xenobiotics is one of the major causes of oxidative stress and apoptosis in the CNS and increases the risk of developing neurodegenerative diseases such as PD [22]. A mechanism by which the cells eliminate xenobiotics is the CYP system, which is involved in the metabolism of most of the drugs and toxins that cross the blood–brain barrier. Among the several isoforms that can be found in this super-family, most of them can be upregulated by at least a few xenobiotics [11]. In our previous publication, we demonstrated that βNF and EtOH are able to induce the expression of two isoforms in SH-SY5Y cells, CYP 2D6 and 2E1 [20]. However, the mechanisms by which CYP isoforms can influence the overall homeostasis of the brain are poorly understood. In this study, we used SH-SY5Y cells to induce the expression of CYPs prior to exposure to MPP^+^, with the aim to study how these two isoforms protect mitochondria against MPP^+^ toxicity. Other publication has also shown that the apoptosis caused by MPP^+^ is mediated by ROS production [23]. We showed that both βNF and EtOH treatments rescue the decrease in cell viability promoted by MPP^+^. Additionally, we presented evidence that the observed neuroprotection is linked to a reduction of ROS formation, restoration of ψ_m,_ and mitochondrial fusion kinetics. Finally, we showed that the toxic effect is avoided before MPP^+^ affects the mitochondrial complex I activity. Taken together, these results suggest that induction of CYPs by βNF and EtOH may contribute to neuroprotection of SH-SY5Y against MPP^+^ toxicity; however, other molecular mechanisms involving neuroprotection pathways not related with CYPs may not be discarded.

The difference in cell viability responses with both concentrations of MPP^+^ outline a possible role of CYPs in MPP^+^ clearance. Indeed, the active site of CYP 2D6 is large enough to allow the entry of MPP^+^ into it, while the active site of 2E1 is smaller, as reported by Gay and colleagues [24]. In this line, a higher presence of CYPs delays the toxicity promoted by MPP^+^, thus observing only late apoptotic or necrotic cells with high concentrations of this toxin. Moreover, our results in mitochondrial functionality are in agreement with those of cell viability. Despite the molecular mechanisms by which CYP 2D6 or 2E1 avoid the toxic effects of MPP^+^ remain unclear, our data are congruent among them and link the rescue in cell viability with restoration of mitochondrial functionality. It is well established that MPP^+^ exerts its toxic effects through inhibition of complex I, disrupting the ATP production and increasing ROS release [25]. According to this mechanistic action, the restoration of mitochondrial homeostasis may be directly related to a decrease in ROS production and a correct functioning of the respiratory chain complex I, as shown in our results.

Consistent with our hypothesis, Mann and colleagues demonstrated that inhibition of CYP 2D6 with quinidine (0.1 µM) increased toxicity and cell death of MPP^+^ in SH-SY5Y cells [26]. Similar results implicating the protective role of this isoform, although in undifferentiated and differentiated PC12 cells, were also reported by Matoh and colleagues, who showed that cells overexpressing *CYP 2D6* cDNA reduced the toxicity and the ROS production promoted by MPP^+^ [27]. Although the neuroprotective role that CYP 2D6 plays in the CNS is still under debate, it may present a dual role in terms of xenobiotic clearance. Indeed, CYP 2D6 have also been involved in the activation of MPTP to MPP^+^, increasing ROS production, altering mitochondrial morphology, and decreasing complex I activity in cultures of Neuro-2A cells expressing CYP 2D6 in mitochondria [28]. More studies are necessary to relate the metabolic activity of CYP 2D6 to MPP^+^ detoxification. It has been shown by our group as well as others that, in neurons, the CYP 2D6 isoform localizes in mitochondria, supporting a possible interaction between both molecules [20,29,30,31].

Our findings are also consistent with other published studies in which the protective role of CYP 2E1 against MPP^+^ toxicity is observed. The role for this isoenzyme in dopaminergic cells is still under study. However, it has been related with MPP^+^ accumulation in vitro rather than with metabolic activity [32]. In mesencephalic cell cultures from CYP 2E1-null mice, the lack of this isoform contributed to the intracellular accumulation of MPP^+^ [33]. The same result was observed in CYP 2E1 knock-out mesencephalic cultures, where the MPP^+^ accumulation was double that in wild-type cells [34]. However, Hao and colleagues showed opposite results in astrocytes, in which the inhibition of CYP 2E1 activity with diallylsulphide avoided the loss of cell viability by MPP^+^ and decreased the ROS production [35].

## 4. Materials and Methods 

### 4.1. Products

βNF (Cat# N3633), EtOH (Cat# E7023), 3-(4,5-dimethylthiazol-2-yl)-2,5-diphenyltetrazolium bromide (MTT; Cat# M2128), 1-methyl-4-phenylpyridinium iodide (MPP^+^; Cat# D048), mitochondria staining kit containing 5,5′,6,6′-tetrachloro-1,1′,3,3′-tetraethyl-benzimidazolocarbocyanine iodide (JC-1; Cat# CS0390), 2′,7′-dichlorofluorescin diacetate (DCFH-DA; Cat# 287810), dimethyl sulfoxide (DMSO; Cat# D8418), and the materials used for cell culture were obtained from Sigma-Aldrich (Milan, Italy). The mitochondrial complex I activity kit (Cat# BVN-K520) was obtained from Vinci-Biochem (Florence, Italy). The Alexa fluor 488™-Annexin V/ propidium iodide (PI) double staining kit (Cat# V13245), the opti-MEM media (Cat# 11058021), and the Lipofectamine^®^ 2000 reagent (Cat# 11668019) were purchased from Life Technologies (Monza, Italy). The mitochondrial reporter (Ds-Red vector) and the plasmid containing the photo-activatable green fluorescent protein (PA-GFP) gene were kindly provided by Professor Richard Youle, NIH (Bethesda, MD, USA).

### 4.2. Cell Culture and Treatments with CYP Inducers and/or MPP^+^

Human neuroblastoma SH-SY5Y cells (ECACC Cat# 94030304) were obtained from Sigma-Aldrich (Milan, Italy) and cultured into polystyrene-coated flasks in RPMI medium (Cat# R8758) supplemented with 10% fetal bovine serum (Cat# F2442), 100 U/mL of penicillin, and 100 μg/mL streptomycin (Cat# P4333). Cells were incubated at 37 °C in 5% CO_2_ and medium was changed two times a week. Once a week, when flasks were at about 80% confluency, the culture was passaged at a ratio of 1:10. Only cultures between passages 3–18 were used.

Cells were seeded in 96-well plates at a concentration of 1.6 × 10^4^ cells/well for MTT assay, mitochondrial complex I activity, and JC-1 experiments, or in 24-well plates at 4.0 × 10^4^ cells/well for ROS detection. In Annexin V/PI and cell cycle experiments, the cells were seeded in 6-well plates at a concentration of 4 × 10^5^ cells/well. After 24 h, the cells were treated as summarized in Table 1, except for mitochondrial complex I activity experiments (see corresponding section for treatment details). In brief, the medium was replaced with fresh medium containing βNF (4 µM) or EtOH (100 mM), and cells were incubated for another 24 h. The medium was then replaced with fresh medium containing the same inducer plus MPP^+^ (0.6 or 1.5 mM) and left for additional 24 hours. Afterwards, cells were used for data acquisition as detailed below. The study was not pre-registered, and no blinding or sample size calculation was performed.

### 4.3. MTT Assay

The transformation of MTT to formazan due to normal metabolic activity, a measure of cell viability, was determined as previously reported [20]. In brief, the culture medium was aspirated, and cells were treated with an MTT solution of 0.5 mg/mL in fresh medium and incubated for 90 min at 37 °C and 5% CO_2_. The solution was then aspirated, and the resulting formazan was dissolved in DMSO. After shaking the plate for 10 min, absorbance was measured by Multiskan GO (Cat# 10588; Thermo Scientific, Milan, Italy) plate reader with SkanIt software version 3.2 (Thermo Scientific, Milan, Italy) at a wavelength of 540 nm. Mean values of absorbance of each treatment from independent experiments were normalized to mean control absorbance values, taken as 100% viability.

### 4.4. Apoptosis Assays

Exposed phosphatidylserine was detected by flow cytometry using the Alexa fluor 488™-Annexin V/PI double staining kit. The staining protocol used for this assay was followed according to manufacturer indications and as previously published [36] with some modifications. In brief, after the treatments described above, the cells were resuspended in 1X annexin-binding buffer and stained with Alexa fluor 488™-Annexin V and PI (100 µg/mL) for 15 min at room temperature. The samples were then read with a BD FACSCalibur Flow Cytometry System (BD Biosciences, San Jose, CA, USA) with CELLQuest Pro software version 3.3 (BD Biosciences, San Jose, CA, USA). Excitation of both fluorescent markers was carried out with a laser at 488 nm, and annexin V and PI were read by using channels FL1 and FL2, respectively. A minimum of 10^4^ cells were collected, and then samples were analyzed with FlowJo™ software vX.0.7 (Becton, Dickinson and Company, Ashland, OR, USA). During the first stages of apoptosis, phosphatidylserine was translocated to the extracellular surface of the membrane and was recognized by annexin V. PI is impermeable to the plasma membrane and only could enter the cells when this was broken during the last stages of apoptosis. Therefore, the population that resulted as a double negative was considered to be healthy. Annexin V positive and PI negative was considered as early apoptotic, double positive was considered as late apoptotic, and annexin V negative and PI positive was considered as a necrotic population.

Cell cycle analysis was also performed to check for apoptotic cells by flow cytometry as previously described by Santulli and colleagues [37]. Briefly, after the treatments detailed before, the cells were fixed with 70% of ice-cold ethanol, treated with RNase (10 mg/mL), stained with PI (2 mg/mL), and incubated for 30 min at room temperature in darkness. Red fluorescence (DNA) was detected through a 563–607 nm band-pass filter using a BD FACSCalibur Flow Cytometry System. A minimum of 10^4^ cells per sample were collected, and the percentage of apoptotic cell accumulated in the sub-G0/G1 peak was calculated by using Cell Quest Pro software version 3.3.

### 4.5. Mitochondrial Fusion Dynamics

The cells were seeded in a glass bottom dish (20 mm) at a concentration of 4 × 10^5^ cells/dish. A day after, cells were transfected with ds-Red (to colocalize mitochondrial) and PA-GFP plasmids (to report fusion dynamics) at a 1:1 proportion (plasmid/plasmid) in opti-MEM medium with lipofectamine transfection reagent at 1:2 ratio (plasmid/lipofectamine). Cells were incubated overnight at 37 °C and 5% CO_2_ and treated with inducers and MPP^+^ (0.6 mM) as detailed in Table 1. Data were acquired as described by Lovy and colleagues [38] with some modifications. In brief, a small region of interest (~10 µm^2^) of the cell was photo-activated at 405 nm with a 40% UV laser. One-minute post photo-activation, cells were photographed every 5 min for 55 min. PA-GFP intensity in pre-photo-activated pictures was taken as 0% intensity, while one-minute post photo-activation was considered as 100% intensity. In healthy cells, intensity decreased over the time in the photo-activated area, indicating high fusion dynamics, while impaired mitochondria retained the fluorescent signal due to a lower fusion rate. Data were acquired with a super-solution Leica motorized inverted microscope (Leica SP8 Gated STED, Leica Microsystems, Ashbourne, Ireland), and analysis was performed with Leica Application Suite X software, version 3.7 (Leica Microsystems, Ashbourne, Ireland).

### 4.6. Mitochondrial Complex I Activity

The Complex I activity experiments were carried out following manufacturer indications and as described by Brown and Brand [39] with some modifications. In brief, cells were treated with βNF (4 µM) or EtOH (100 mM) for 48 hours to promote induction of CYPs. After incubation, the medium was replaced for assay buffer containing KCN (100 mM), fatty acid free-BSA (1:20), and bovine heart mitochondria (1:50). Then, test compound (MPP^+^ 1.5 mM), positive control (rotenone, 1 µM) or vehicle, diluted in assay buffer, were added to each well. Finally, buffer containing NADH reagent (1:23) and ubiquinone reagent (1:34) was added to each well. Immediately after, data were acquired in a plate reader by measuring the absorbance at 340 nm every 30 s during 15 min at room temperature. Absorbance was plotted versus time, and slope was calculated. The complex I activity (%) was determined by dividing the slope of each sample by the control slope value. 

### 4.7. Mitochondrial Electrochemical Potential Gradient

Changes in the ψ_m_ were detected with a carbocyanine dye JC-1 kit following manufacturer’s indications. Under normal ψ_m_, JC-1 accumulated inside mitochondria, forming aggregates that emitted a red fluorescence; under depolarized ψ_m_, JC-1 diffused to the cytoplasm and formed monomers that emitted a green fluorescence. After treating the cells as described in Table 1, a Fluoroskan Ascent fluorimeter (Cat# 1506450; Thermo Labsystems, Milan, Italy) was used to measure the red- (aggregated JC-1, intact mitochondria, 525 nm excitation, 590 nm emission) or the green-fluorescence (monomeric JC-1, disrupted mitochondria, 490 nm excitation, 530 nm emission). Mitochondrial membrane depolarization was indicated by a decrease in the red/green fluorescence intensity ratio. Valinomycin (0.2 ng/μL for 20 min) was used as a positive control. 

### 4.8. ROS Detection

ROS generation was assessed as previously described by Brizi and colleagues [40] with some modifications. In brief, after treatments described in Table 1, cells were rinsed with phosphate buffered saline (PBS) and loaded with 10 μM 2′,7′-dichlorofluorescin diacetate (DCFDA) for 15 min at 37 °C, then washed, centrifuged at 13,000× *g* for 5 min, and re-suspended in 0.7 mL of phosphate buffered saline (Cat# P5493; Sigma-Aldrich, Milan, Italy). The intracellular fluorescence (504 nm excitation, 529 nm emission) was acquired with a Fluoroskan Ascent fluorimeter, and data were normalized to mg of cellular protein of the samples and expressed as percent of untreated control cells.

### 4.9. Statistical Analysis

Data were reported as mean ± SEM and analyzed for statistical significance by two-way ANOVA followed by Tukey’s multiple comparison test with IBM SPSS Statistics for windows, version 26 (IBM Corp., Armonk, N.Y., USA). No exclusion criteria were pre-determined. No normality test to analyze the data distribution was performed. Outliers that were two standards deviation away from the mean were excluded from the analysis, since they likely represent technical errors.

## 5. Conclusions

We propose that the neuroprotection mediated by βNF and EtOH may be carried out by the metabolic activity of CYP 2D6 in mitochondria and by the role of CYP 2E1 in the efflux and the accumulation of MPP^+^. However, other pathways promoted by βNF, EtOH, and independent from CYP induction should not be discarded. Taken together, these results support a possible role of CYP 2D6 and 2E1 as a potential neuroprotective system against xenobiotic insult in undifferentiated SH-SY5Y cells. Further research is needed to establish this CYP induction model in other cell lines and primary cultures and to confirm a direct implication of CYP in MPP^+^ neuroprotection, thus the impact that these isoforms have in the metabolism of other xenobiotics bring new insights for future therapeutic approaches. 

## Figures and Tables

**Table 1:** Experimental protocol for drug treatments. The day after plating the cells, they were treated for 24 h with fresh medium containing Cytochrome P450 (CYP) inducers (see column 0–24). Afterward, the medium was replaced with fresh medium containing CYP inducers, the neurotoxin 1-methyl-4-phenylpyridinium (MPP^+)^ or a combination of both (see column 24–48).

Sample/Group Name	0–24 h	24–48 h
Control	Medium	Medium
βNF	βNF (4 μM)	βNF (4 μM)
EtOH	EtOH (100 mM)	EtOH (100 mM)
MPP^+^ (0.6 mM)	Medium	MPP^+^ (0.6 mM)
MPP^+^ (1.5 mM)	Medium	MPP^+^ (1.5 mM)
βNF + MPP^+^ (0.6 mM)	βNF (4 μM)	βNF (4 μM) + MPP^+^ (0.6 mM)
βNF + MPP^+^ (1.5 mM)	βNF (4 μM)	βNF (4 μM) + MPP^+^ (1.5 mM)
EtOH + MPP^+^ (0.6 mM)	EtOH (100 mM)	EtOH (100 mM) + MPP^+^ (0.6 mM)
EtOH + MPP^+^ (1.5 mM)	EtOH (100 mM)	EtOH (100 mM) + MPP^+^ (1.5 mM)

## Supplementary Materials

Supplementary materials can be found at https://www.mdpi.com/1422-0067/21/11/3955/s1.

## Abbreviations

CNS	Central Nervous System
CYP	Cytochrome P450
DCFH	2′,7′-dichlorofluorescein
DCFH-DA	2′,7′-dichlorofluorescin diacetate
EtOH	Ethanol
JC-1	5,5′,6,6′-tetrachloro-1,1′,3,3′-tetraethyl-benzimidazolocarbocyanine iodide
MPP^+^	1-methyl-4-phenylpyridinium
MTT	3-(4,5-dimethylthiazol-2-yl)-2,5-diphenyltetrazolium bromide
PA-GFP	Photo-activatable green fluorescent protein
PD	Parkinson’s disease
PI	Propidium Iodide
ROS	Reactive oxygen species
βNF	β-naphthoflavone
ψ_m_	Mitochondrial membrane potential
