# Supplementary material for: β-Naphthoflavone and Ethanol Reverse Mitochondrial Dysfunction in A Parkinsonian Model of Neurodegeneration
Source: Int J Mol Sci. 2020 May 31;21(11):3955. doi: 10.3390/ijms21113955 (PMC7312836; doi:10.3390/ijms21113955)
Supplement: Supplementary file 1 [file ijms-21-03955-s001.pdf]

Article title: Cytochrome P450 induction reverses mitochondrial dysfunction in a parkinsonian model of neurodegeneration

Journal Name: International Journal of Molecular Sciences

Authors: Jesus Fernandez-Abascal[1ǂ*], Elda Chiaino[1], Maria Frosini[1], Gavin P Davey[2] and Massimo Valoti[1]

[1] Dipartimento di Scienze della Vita, Università di Siena, Italia; maria.frosini@unisi.it (M.F.); massimo.valoti@unisi.it (M.V.); chiaino@student.unisi.it (E.C.).

[2] School of Biochemistry and Immunology, Trinity College Dublin, Dublin, Ireland; gdavey@tcd.ie (G.P.D.).

ǂ Present address: Department of Physiology and Biophysics, University of Miami, Miller School of Medicine, 1600 NW 10th Ave, Miami, FL 33136, USA.

* Correspondence: Dr. Jesus Fernandez-Abascal jxf952@med.miami.edu (J.F.A)

Supplementary material 1

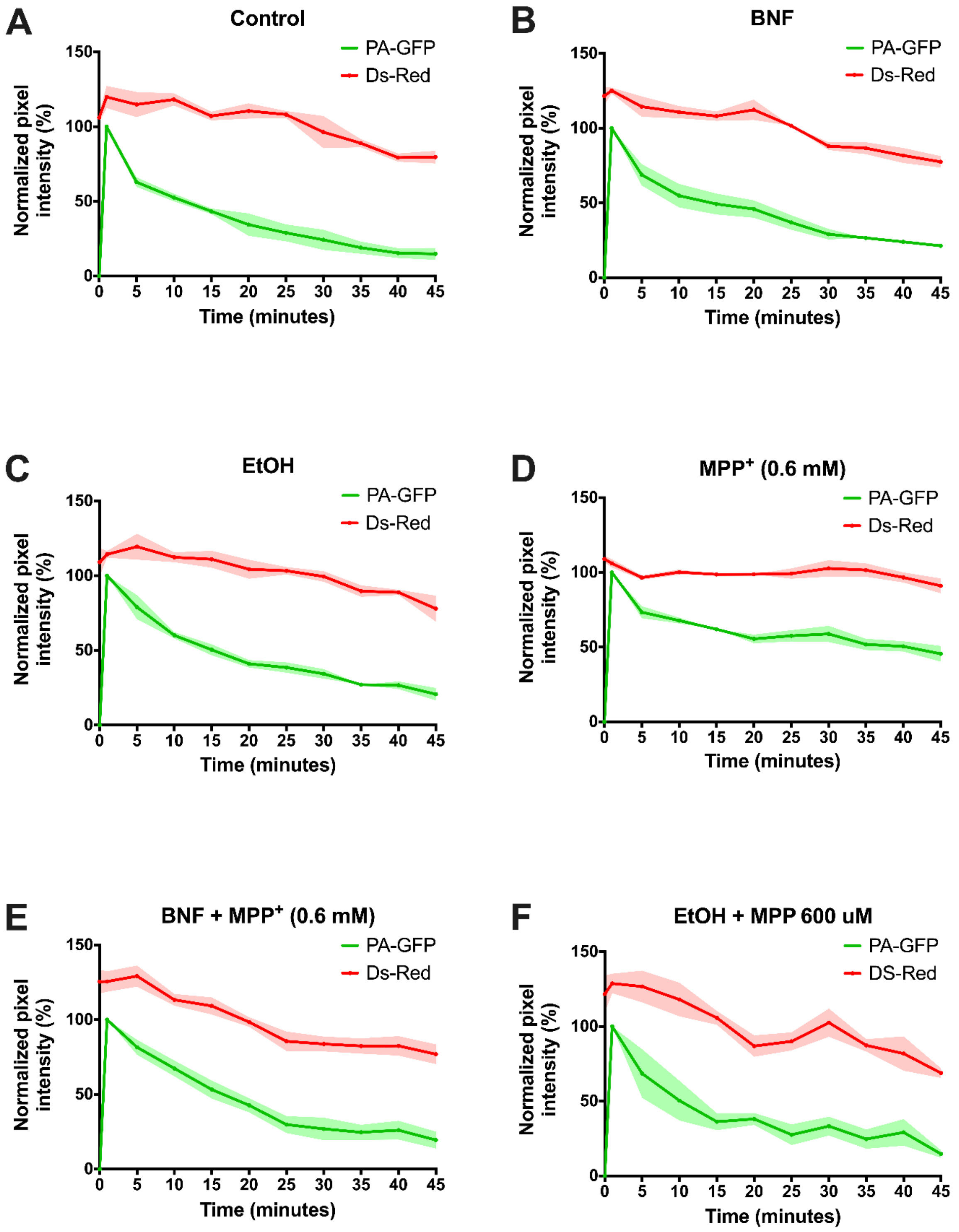

Supplementary material 1. Mitochondrial fusion dynamics in SH-SY5Y cells. PA-GFP is gradually spread in mitochondria over the 45 minutes of assay. The intensity of fluorescence in the photo-activated area consistently decreases until 15-20% at minute 45. Ds-Red was used as a mitochondrial marker and calibrator. Data represent the normalized pixel intensity (%) ± SEM of at least 3 independent experiments.
